# Synergistic delivery of 5-fluorouracil and curcumin using human serum albumin-coated iron oxide nanoparticles by folic acid targeting

**DOI:** 10.1007/s40204-018-0104-3

**Published:** 2018-12-18

**Authors:** Chinmay G. Hiremath, Mahadevappa Y. Kariduraganavar, Murigendra B. Hiremath

**Affiliations:** 1 Department of Biotechnology and Microbiology, Karnatak University, Dharwad, Karnataka 580003 India; 2 Department of Chemistry, Karnatak University, Dharwad, Karnataka 580003 India

**Keywords:** Human serum albumin, Magnetic nanoparticles, 5-Fluorouracil, Curcumin, Folic acid

## Abstract

Human serum albumin is the most abundant protein in plasma with the ability to bind to a variety of drug molecules. Magnetic nanoparticles are being extensively used in drug delivery due to its intrinsic magnetic properties. In this work, we have synthesized human serum albumin-coated citrate-functionalized iron oxide nanoparticles by CDI coupling. Furthermore, folic acid was decorated on human serum albumin by EDC and NHS coupling to confer targetability. Two cytotoxic drugs 5-fluorouracil (5FU) and curcumin were co-delivered. Wherein, the former is an anticancer agent and latter is a drug resistance depressor of former. The nanoparticles showed good aqueous dispersibility with a zeta potential of − 49.1 mV and magnetic core size in the range of 10–15 nm, thus exhibiting good magnetic property with magnetic saturation of 33.59 emu/g. Controlled drug release behavior was noticed in both drugs with faster release profile of 5FU. Nanoparticles also showed good cytotoxicity with lower IC_50_ values in the presence of magnetic field. The contrasting difference was noticed in folic acid-decorated and non-decorated composites, similarly in the presence of magnetic field where cell uptake was enhanced.

## Introduction

Despite the advancement in cancer treatment modalities, such as surgical intervention, radiation, and chemotherapeutic drugs, cancer yet remains one of the world’s most catastrophic diseases; with more than 10 million new cases every year (Hamzehalipour Almaki et al. 2017). Extermination of healthy cells and systemic toxicity lower the quality of patient’s life. Inability to administer therapeutic moieties to acquire selectivity of the desired targets with marginal or no collateral damage has largely accounted for the discrepancy (Peer et al. 2007).

The growth of nanotechnology has unfolded a new paradigm of possibilities in the development of medical sciences like drug delivery field, leading to the development of new drug carrier composites in the nanometer size (Lohcharoenkal et al. 2014; Roco 2011). Nanoparticles, comprising active pharmaceutical ingredients with biocompatible materials in nanometer size, have shown enhanced anticancer property, improved pharmacokinetics, pharmacodynamics, and active intracellular delivery (Lomis et al. 2016). Concurrently, nanoparticles (NPs) are being developed as drug carriers. Thanks to careful nanostructure construction (tailored drug release characteristics, low immunogenicity, etc.), yielding improved treatment efficacy and reduction of unwanted side effects (Veiseh et al. 2010).

Among myriad non-therapeutic constructs for effective and safe therapeutics, protein-based carriers are ideal because of: greater shelf life, amphiphilicity, amenability for surface modification, biodegradability, and ease of ligand bioconjugation (Tarhini et al. 2017). HAS is a major plasma protein involved in the maintenance of osmotic pressure and transport of nutrients to the cells. Its high aqueous solubility (up to 40% w/v) at pH 7.4 and its binding ability to a wide variety of drugs has made it ideal for drug delivery application (Lohcharoenkal et al. 2014). Hence, HAS is used in this work. Many drugs and endogenous molecules are known to bind to albumin. These properties, as well as its preferential uptake in tumor and inflamed tissue, have made it an ideal material for drug delivery (Elzoghby et al. 2012; Kratz 2008).

Magnetic nanoparticles (MNP) like Fe_3_O_4_ due to its intrinsic magnetic properties have attracted curiosity that enables magnetic targeting, magnetic resonance imaging, magnetic hyperthermia, etc. In addition to these physicochemical profiles, MNP can be easily tailored for a functional group, inexpensive production, and possesses non-offensive toxicity profile (Lu et al. 2007; Veiseh et al. 2010). Moreover, Fe_3_O_4_-based products like Feraheme^®^, Feridex I.V.^®^, and Gastromark^®^ have already gained approval for biomedical applications (Revia and Zhang 2016). Upon metabolism, iron ions are added to the body’s iron stores and eventually incorporated by erythrocytes allowing for their safe use in vivo (Sun et al. 2008).

5-Fluorouracil (5FU), an anti-metabolite drug, has been attributed to incorporate fluoronucleotides in place of nucleotides that inhibits the nucleic acid synthetic enzyme thymidylate synthase (TS), hence, exhibiting cytotoxicity. It is being used in the treatment of a range of cancers namely colorectal cancer, breast cancers, and cancers of the aerodigestive tract. However, dihydropyrimidine dehydrogenase (DPD), an enzyme abundantly expressed in liver, catabolizes more than 80% of administered 5FU to dihydrofluorouracil (DHFU) (Longley et al. 2003). Furthermore, 5FU also up-regulates several survival signals including NF-kB and Akt. NF-kB pathway is a major downstream effect or pathway leading to chemoresistance (Vinod et al. 2013).

Curcumin (CUR), a natural polyphenol from the root of *Curcuma longa* Linn, is a pharmacologically safe. Although hydrophobicis yet potent anticancer molecule against a variety of cancers including breast cancer (Yu et al. 2014; Kakran et al. 2012; Rachmawati et al. 2013). Many mechanisms are involved in the biological activities of CUR including NF-kB, IkBa kinase, Akt, activator protein-1, mitogen-activated protein kinases (MAPK), cyclooxygenase-2 and 5-lipoxygenases, inducible nitric-oxide synthase, urinary plasminogen activator, tumor necrosis factor, chemokines, and cell cycle machinery which have been suggested as the targets of CUR (Choi et al. 2008; Li and Zhang 2014). Curcumin downregulates NF-kB, both directly or via TS pathway, thereby circumventing 5FU resistance. Thus, down regulation of NF-kB by chemopreventives is an effective mechanism to tackle drug resistance (Vinod et al. 2013). However, the limited clinical utility of curcumin is due to its poor solubility and unstable at neutral and basic pH (Saengkrit et al. 2014).

Folic acid (FA), vitamin B9, is vital for the maintenance and proliferation of all the cells. Folic acid receptors are overexpressed on the surface of many human tumor cells, including ovarian, lung, breast, endometrial, renal, and colon cancers (Sun et al. 2006). Significant up-regulation of the folate receptor on tumor tissue has led to the hypothesis that folate-linked therapeutic agents might display reduced off-site toxicity and enhanced potency against tumor cells compared to non-targeted drugs (Xia and Low 2010). FA and FA conjugates can bind to the FRs with high affinity and enter cells by receptor-mediated endocytosis, so the FA-modified drug delivery vectors can transfer the therapeutic agents to tumor cells that exhibit amplified foliate receptor expression. In case of normal cells, FRs’ expression is much lower (Lin et al. 2016).

In this work, we have synthesized folic acid-decorated human serum albumin-coated Fe_3_O_4_ nanoparticles (C-MNP-HSA-FA) for synergistic delivery of 5-fluorouracil and curcumin for the treatment of breast cancer. This work attempts to entrap 5FU to circumvent its metabolism and further lower chemoresistance of 5FU using CUR. HSA confers CUR solubility and higher bioavailability.

## Materials and methods

### Materials

Anhydrous iron (III) chloride (FeCl_3_), iron (II) chloride tetrahydrate (FeCl_2_·4H_2_O) 99%, ammonium hydroxide (30–33% M), Tween-80, folic acid (FA), *N*,*N*′-carbonyldiimidazole (CDI), (1-ethyl-3-(3-dimethylaminopropyl)carbodiimide (EDC), and *N*-hydroxysuccinamide (NHS) were purchased from Sigma-Aldrich. Oleic acid and sodium citrate were purchased from TCI chemicals. 5-Fluorouracil and curcumin were purchased from MOLchem. Nitrogen-purged Mili-Q was used in all the steps involved in the synthesis and formulation of magnetic nanoparticles.

### Synthesis of iron oxide magnetic nanoparticles (MNP)

Iron oxide nanoparticles were prepared by alkaline co-precipitation method (Yallapu et al. 2011; Ahn et al. 2012). Briefly, to the solutions of 0.1 M Fe^3+^(100 mL) and Fe^2+^(50 mL) at 80 °C 10 mL of 25%, ammonia solution was added dropwise for 1 min with constant stirring at 6000 rpm. A 50 mL of 0.005 M trisodium citrate was added and stirred for 30 min under an inert atmosphere (N_2_). Thus, obtained citric acid-coated Fe_3_O_4_ (C-MNP) were washed multiple times by magnetic decantation using milliQ water and stored at 4 °C.

### Coating of magnetic nanoparticles with HSA by CDI coupling

C-MNP of 100 mg was suspended in MilliQ water by sonication. C-MNP suspensions were washed with water and DMSO ratio (1:4, 2:3, 3:2, and 4:1 v/v) and finally washed three times with anhydrous DMSO to eliminate traces of water. To the anhydrous C-MNP, 1 g of CDI was added. The reaction mixture as was allowed to stir at room temperature for 6 h. The C-MNP suspension was washed with DMSO to obtain CDI-free MNP CDI adducts.

C-MNP CDI adduct was added dropwise to 100 mL of 1 mg/mL of a buffered solution of HSA. The reaction mixture was allowed to stir for 12 h. The unreacted HSA was washed with phosphate buffer saline by centrifugation to obtain serum albumin-coated magnetic nanoparticles (C-MNP-HSA).

### FA conjugation to C-MNP-HSA

The FA was coupled covalently on the surface of C-MNP-HSA nanoparticles using zero-length crosslinkers, EDC and NHS (Yang et al. 2014). To 5 mg of folic acid, dissolved in 10 mL of dry DMSO, 15 mg of NHS and 30 mg of EDC were added under vigorous stirring. The reaction mixture was allowed to stir for 1 h in the dark at room temperature to obtain activated FA.

A 50 mg of C-MNP- HSA was dissolved in 100 mL of carbonate buffer solution (pH 9.8, 0.05 M). To this, 5 mL of activated folic acid in DMSO was added dropwise. The reaction was allowed to stir 12 h in the dark at room temperature. Folic acid-decorated nanoparticles were washed multiple times by centrifugation.

### Physical and chemical characterization of nanoparticles

Fourier transform infrared spectroscopy (FTIR) of MNP, C-MNP, C-MNP-HSA, and C-MNP-HSA-FA was recorded using Thermo Nicolet 8700, USA in 4000–500 cm^−1^ range, with KBr as the sample holder.

The hydrodynamic particle-size measurements and surface charge of MNP, C-MNP, C-MNP-HSA, and C-MNP-HSA-FA were measured by dynamic light scattering using Horiba SZ-100 particle-size analyzer. The size measurement was carried out in polystyrene cuvettes and zeta potential was measured using a graphite electrode polystyrene cuvette. All the samples were of concentration 1 mg/10 mL, at 25 °C temperature, and pH 7.4.

Particles were analyzed by Philips/FEI Inc., Briarcliff, Manor, NY, transmission electron microscope (TEM). Sample preparation was carried out by a drop of an aqueous dispersion of C-MNP-HSA-FA nanoparticles on a carbon-coated copper TEM grid and was allowed to air dry.

Thermogravimetric analysis (TGA) of MNP, C-MNP, and C-MNP-HSA-FA was recorded using SDT Q600, USA. A 5 mg sample was placed in the aluminum sample holder and heated at a ramp rate of 10 °C/min to 600 °C under nitrogen environment.

Magnetic properties of MNP, C-MNP, and C-MNP-HSA-FA were measured using Lakeshore, Model 7407, USA, vibrating sample magnetometer (VSM) at 15 kOe applied magnetic field at room temperature.

### Drug entrapment efficiency and drug release study

In drug-loading procedure, 5 mg of 5FU and 5 mg of CUR were dissolved in 5 mL of DMSO and were added dropwise to 50 mg aqueous dispersion of C-MNP-HSA-FA. The mixture was allowed to stir for 6 h at room temperature. 5FU and CUR-loaded C-MNP-HSA-FA (5FU-CUR-C-MNP-HSA-FA) was separated by centrifugation.

The drug loading (DL) and entrapment efficiency (EE) of 5FU and CUR in C-MNP-HSA-FA were determined using the following equations:

1 $${\text{DL }}(\% ) = \frac{\text{Weight of drug in nanoparticles}}{\text{Weight of nanoparticle}} \times 100,$$

2 $${\text{EE }} \left( \% \right) = \frac{\text{Weight of drug in nanoparticles}}{\text{Weight of drug loaded}} \times 100.$$

The drug release was performed by suspending 10 mg of 5FU-CUR-MNP-HSA-FA NPs in 50 mL of phosphate buffer solution (pH 7.4) with 0.05% (w/v) of Tween^®^80 at 37 °C. At specific time intervals, 1 mL of release buffer was aspirated and replaced by fresh phosphate buffer solution. The concentration of 5FU and CUR was determined using a Hitachi U-2800 UV–Vis spectrophotometer at a wavelength of 241 nm and 421 nm, respectively.

### In vitro cytotoxicity assay of drug-loaded nanocarriers

The cytotoxic properties of 5FU, CUR, C-MNP-HSA-FA, 5FU-CUR-C-MNP- HSA, 5FU-CUR-C-MNP-HSA-FA, and 5FU-CUR-C-MNP-HSA-FA under magnetic field (5FU-CUR-C-MNP- HSA-FA-Mag) was determined by MTT cell viability assay. MCF-7 cells were seeded onto 96-well plates at 5000 cells per well. After 24 h, the cells were treated with different concentrations (0.01, 0.1, 1, 10, and 100 μg/mL) of 5FU, CUR, C-MNP- HSA -FA, 5FU-CUR-C-MNP-HSA, 5FU-CUR-C-MNP-HSA-FA, and 5FU-CUR–MNP-HSA-FA-Mag. For 5FU-CUR-MNP-HSA-FA-Mag, cells were cultivated under 0.05 T magnet placed beneath the wells. After the incubation, the cells were washed multiple times with DPBS and replaced with fresh growth medium. To assess the cell viability, 50 µL dye solution was added to 100 µL of the medium in each well. The formazan crystals thus formed after incubation for 4 h at 37 °C in 5% CO_2_ atmosphere was solubilized in 100 µL of DMSO, and the plates were gently shaken to solubilize the formed formazan. The absorbance was then measured with a spectrophotometer at 540 nm.

### Cellular uptake

Quantitative uptake of C-MNP-HAS and C-MNP-HSA-FA in the presence and absence of magnetic field was evaluated by measuring iron content in the cell by atomic absorption spectroscopy (AAS) (Prijic et al. 2010; Dinda et al. 2012). MCF-7 were seeded in 6-well plates, upon reaching 90% confluence, the supernatant was discarded and replaced by 2 mL of nanocomposite suspension in culture medium (iron content of 200 µg/mL). Plates were then placed in a 37 °C, 5% CO_2_ incubator for the duration of 60 and 240 min. After incubation, cells were trypsinized and washed with DPBS three times to ensure the complete removal of media and leftover nanoparticles. The cell pellets of each well were then digested by adding 1 mL of HCl. After 24 h, dilution at 1:4 was made and the iron content of the cell lysate was analyzed by AAS.

### Statistics

Drug release study, MTT assay, and cellular uptake data were collected in triplicates and analyzed statistically using SPSS 20 for standard mean and standard deviation.

## Results and discussion

**Fig. 1:**
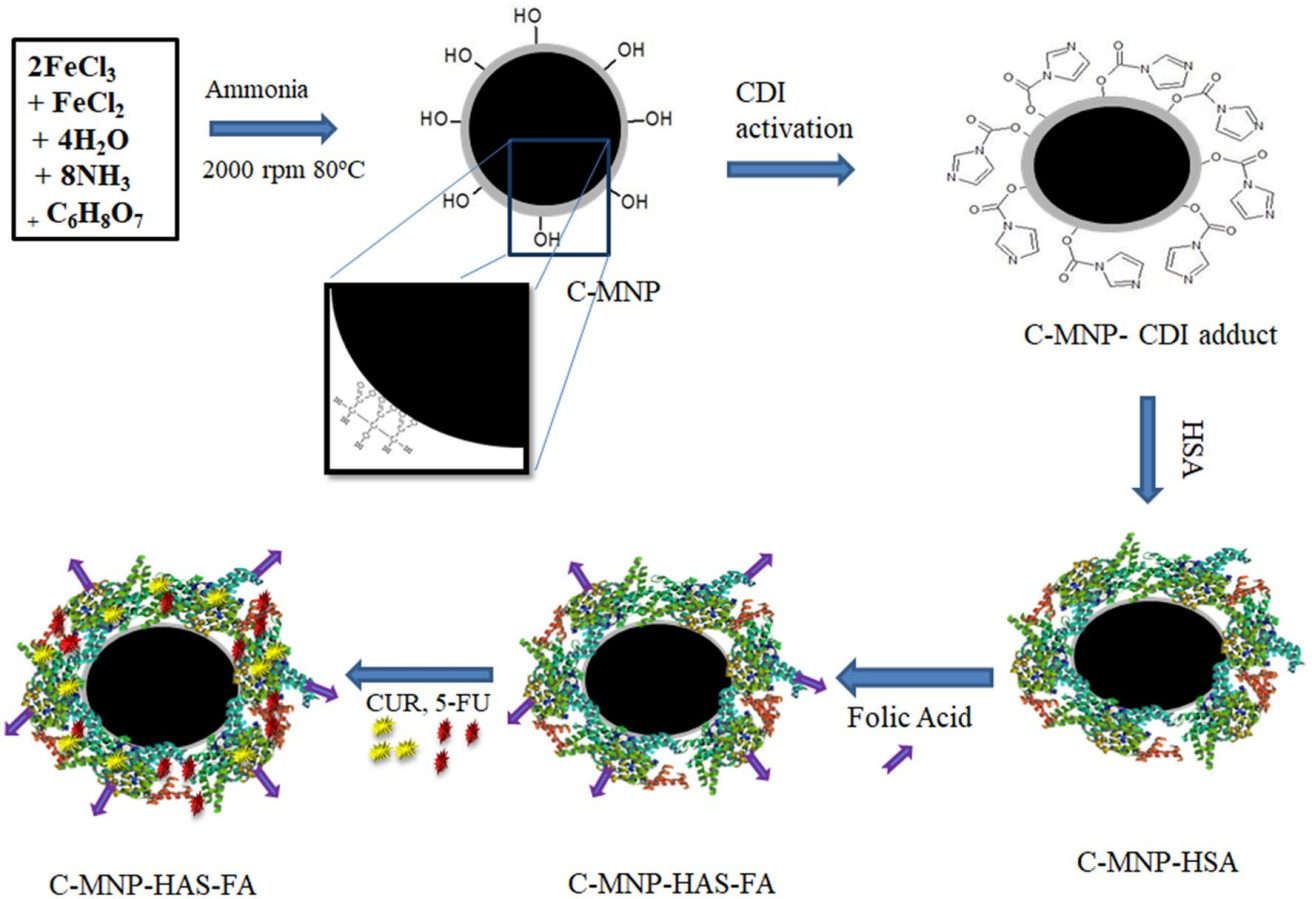
Graphical abstract depicting synthesis of 5FU-CUR-C-MNP-HSA-FA. *MNP* magnetic Fe_3_O_4_ nanoparticles, *C-MNP* citric acid-coated MNP, *5FU-CUR-C-MNP-HSA-FA* 5-fluorouracil and curcumin. Entrapment in folic acid-decorated albumin-coated citrate-modified magnetic nanoparticles

The magnetic nanoparticles are carving a niche into the biomedical applications since a couple of decades (Tran and Webster, 2010). Size and proper functionalization are of utmost importance for its application (Ali et al. 2016). Synthesis of 5FU-CUR-C-MNP-HSA-FA is as depicted in Fig. 1. Alkaline precipitation method was employed for the synthesis of Fe_3_O_4_ nanoparticles. Addition of ammonia to iron salts changed color from orange to black. Nanoparticles were synthesized by alkaline co-precipitation method and coated with citric acid. Citric acid apart from functionalization confers good dispersibility and also acts as a capping agent. Citric acid-coated Fe_3_O_4_ was previously found to have a good biomedical application like MRI contrast agent and in hyperthermia (Andreas et al. 2012). The nanoparticles showed good aqueous dispersity. The obtained nanoparticles were washed by magnetic decantation. HSA, protein found abundantly in human blood plasma, can tolerate pH and temperature constraints. Multiple functional groups in HSA provide an opposite for binding of hydrophobic and hydrophilic drug molecules. Hence, we have coated iron oxide nanoparticles with HSA. HSA was immobilized on citrate-functionalized MNP by CDI coupling, followed by folic acid decoration using zero-length crosslinkers. FA conjugation confers nanocomposites targetability to cancer cells (Meng et al. 2011).

### Fourier transform infrared spectra

**Fig. 2:**
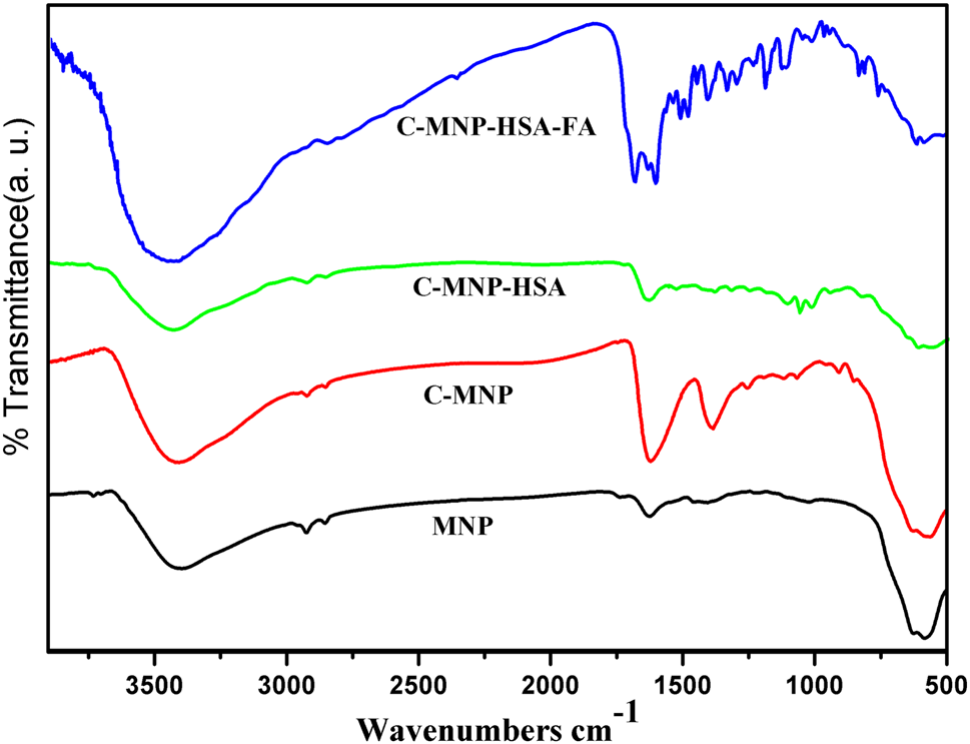
FTIR spectra of MNP, C-MNP, C-MNP-HSA, and C-MNP-HSA-FA. *MNP* magnetic Fe_3_O_4_ nanoparticles, *C-MNP* citric acid-coated MNP, *C-MNP-HSA* albumin-coated citrate-modified magnetic nanoparticles, *C-MNP-HSA-FA* folic acid-decorated albumin-coated citrate-modified magnetic nanoparticles

FTIR spectra of MNP, C-MNP, C-MNP-HSA, and C-MNP-HSA-FA are as shown in Fig. 2. MNP shows a prominent peak of Fe–O vibrations at 576 cm^−1^ which corresponds to vibration of the Fe–O bonds in the crystalline lattice of Fe_3_O_4_ (Yang et al. 2014). The peaks at 1620 and 1384 cm^−1^ in C-MNP spectra correspond to asymmetric and symmetric stretching of carboxyl group confirming the presence of citric acid; the shift in the carboxyl group stretch may be due to chemisorption of carboxyl group (Saraswathy et al. 2014). Pure HSA exhibits a characteristic spectral band at 1634 (amide I) and 1528 (amide II). All these bands are present in C-MNP-HAS confirming attachment of HSA to C-MNP indicating the confirmation of HAS immobilization. A band appears around 1607 cm^−1^ that we assign to the C_4_=N_3_-stretching mode of the pterin ring of FA. The FA peaks at 1542 and 1514 cm^−1^ are from the C=C stretches of the heterocyclic ring and heterocyclic ring breathing in PCA (Li et al. 2012). FA displays similar peaks in C-MNP-HSA-FA which indicates successful conjugation of FA to C-MNP-HAS.

### Size and zeta potential

**Fig. 3:**
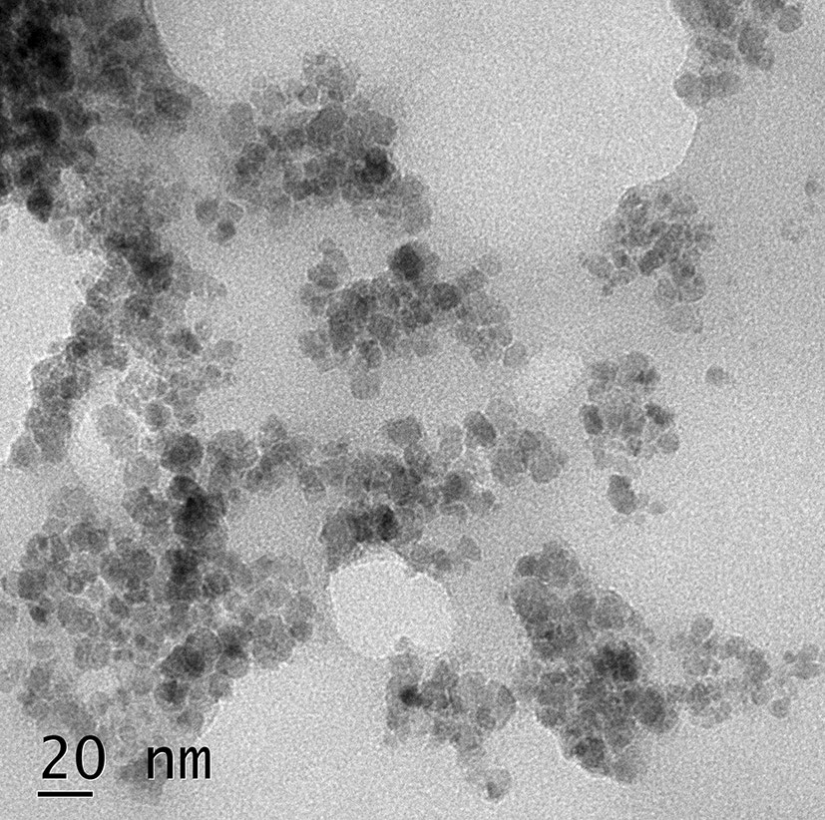
Transmission electron micrographs of C-MNP-HSA-FA

The particle size and zeta potential of MNP, C-MNP, C-MNP-HAS, and C-MNP-HSA-FA measured are as shown in Table 1. Size of C-MNP-HSA-FA was found 108.4 nm with − 49.1 mV zeta potential. There is an increase in size with a coating of citric acid and HSA. The anomalously bigger size of MNP in contrast to C-MNP is due to agglomeration of particles. The larger size of MNP may be due to lack of capping agent and low zeta potential (Granata et al. 2016). This can be backed up by low zeta potential and a high polydispersity index of MNP. Zeta potential is an overall surface charge of the nanoparticle; it is a degree of repulsion between particles of similarly charged colloids in suspension. Large negative and positive zeta potential values in the suspension diminish the aggregation behavior of the particles (Arya et al. 2011). Samples C-MNP-HAS and C-MNP-HAS-FA bestow zeta potential of − 50.9 mV and − 49.1 mV, respectively. These values are well beyond ± 30 mV, indicating high colloidal stability of the nanoparticles (Bhattacharjee 2016). C-MNP particle-size distribution is very narrow with a polydispersity index of 0.190. The contrasting drop in the size of the nanoparticle to uncoated MNP is due to a citric acid coating which confers MNP a good dispersibility. C-MNP-HSA showed an increase in size which can be accredited to protein coat. No significant change in size is observed in size upon functionalization with FA. A slight reduction in zeta potential may be due to the coating of positively charged FA on BSA with a negative charge. Transmission electron microscopy images measure mean size of the iron core as 10 nm (Fig. 3).

**Table 1:** Size and zeta potential analysis of MNP, C-MNP, C-MNP-HAS, and C-MNP-HAS-FA

Sample	Mean particle size (nm)	Zeta potential	Polydispersity index
1. NMP	124.0	− 2.1	2.313
2. C-MNP	51.2	− 40.2	0.190
3. C-MNP-HSA	107.9	− 50.9	1.844
4. C-MNP-HSA-FA	108.4	− 49.1	1.844

*MNP* magnetic Fe_3_O_4_ nanoparticles, *C-MNP* citric acid-coated MNP, *C-MNP-HSA* albumin-coated citrate-modified magnetic nanoparticles, *C-MNP-HSA-FA* folic acid-decorated albumin-coated citrate-modified magnetic nanoparticles

### Thermogravimetric analysis

**Fig. 4:**
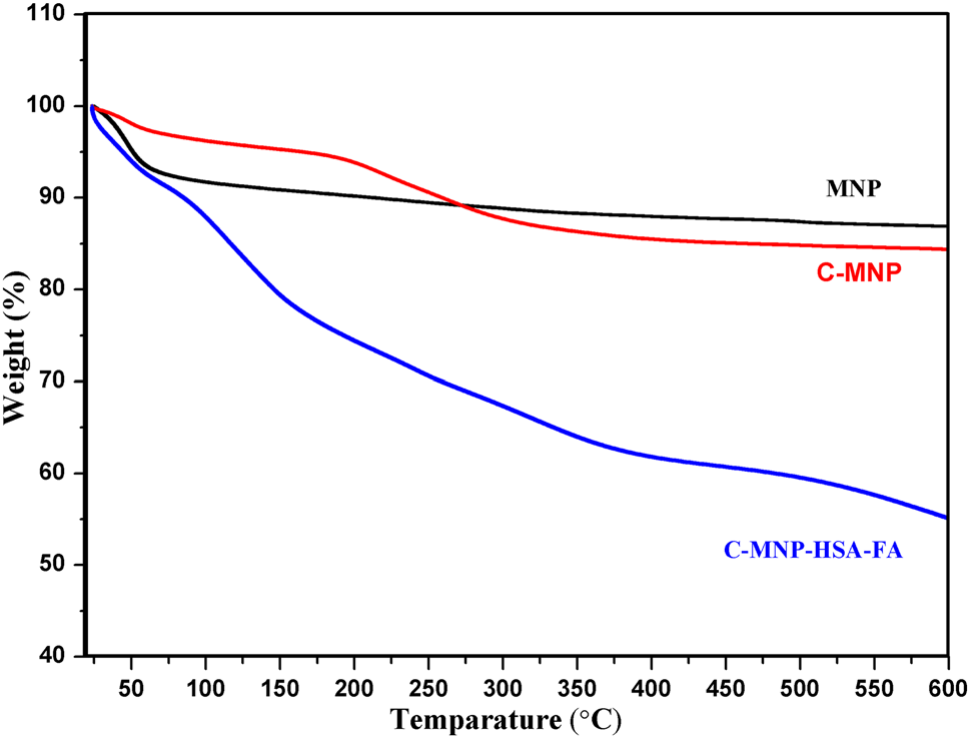
Thermograms of MNP, C-MNP, and C-MNP-HSA-FA. *MNP* magnetic Fe_3_O_4_ nanoparticles, *C-MNP* citric acid-coated Magnetic Nanoparticles, *C-MNP-HSA-FA* folic acid-decorated albumin-coated citrate-modified magnetic nanoparticles

Thermal decomposition graph of MNP, C-MNP, and C-MNP-HSA-FA is as shown in Fig. 4. Thermogravimetric analysis shows weight loss with an increase in temperature. According to the graph, the observed loss of mass at around 90 °C can be accredited to moisture loss. The weight loss witnessed at 190 °C by C-MNP is due to decomposition of citrate moiety in C-MNP. Since the total loss of 10% was observed in the case of C-MNP, it may indicate the percentage composition of citric acid. In the case of C-MNP-HSA-FA apart from moisture loss of 12.12%, 32.75% loss was noted. This is due to decomposition of HSA and FA, i.e., total of 32.75% is the protein component in the nanoparticles.

### Vibrating sample magnetometry

**Fig. 5:**
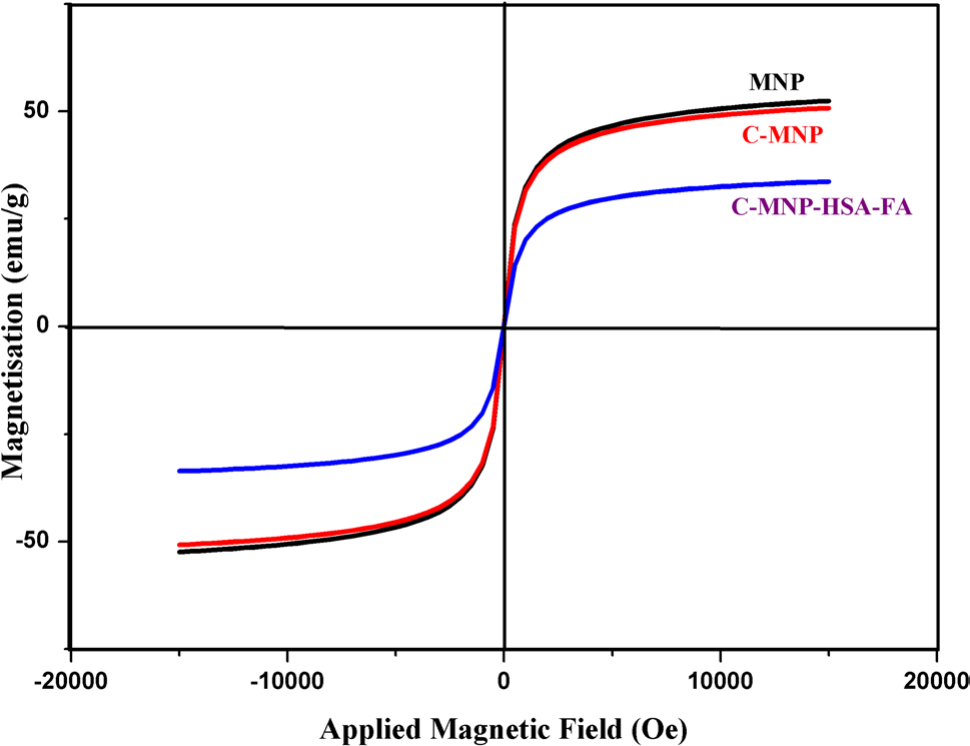
Magnetic hysteresis analysis of MNP, C-MNP, and C-MNP-HSA-FA. *MNP* magnetic Fe_3_O_4_ nanoparticles, *C-MNP* citric acid-coated MNP, *C-MNP-HSA-FA* folic acid-decorated albumin-coated citrate-modified magnetic nanoparticles

For effective use as magnetically targeted drug delivery systems, nanoparticles should exhibit superparamagnetism with high saturation magnetization value. Superparamagnetism is due to randomization of magnetic moment at room temperature. It is characteristic magnetization, where nanoparticles are magnetized in the presence of magnetic field and immediately demagnetized when a magnetic field is turned off (Cole et al. 2011). Magnetic saturation (Ms) values of MNP, C-MNP, and C-MNP- HSA-FA were found to be 52.04 emu/g, 50.6 emu/g, and 33.59 emu/g, respectively. MNP, C-MNP, and C-MNP-HSA-FA samples showed negligible coercion of 0.51551 G, 0.5122 G, and 0.24485 G, respectively (Fig. 5). The magnetometry shows no obvious signs of hysteresis, i.e., neither magnetic remanence nor coercion. This confirms the superparamagnetic characteristic of MNP, C-MNP, and C-MNP-HSA-FA. Not much of decrease in Ms was noted in case of C-MNP. However, Ms post-coating with HSA was reduced due to the coating of HAS, which reduces the weight ratio of the iron core in nanoparticles (Yu et al. 2013). Since C-MNP-HSA-FA nanoparticles are superparamagnetic and size of the magnetic core is 10 nm, they can be ideal for use in drug delivery (Wahajuddin 2012).

### Entrapment efficiency and in vitro release of drugs

**Fig. 6:**
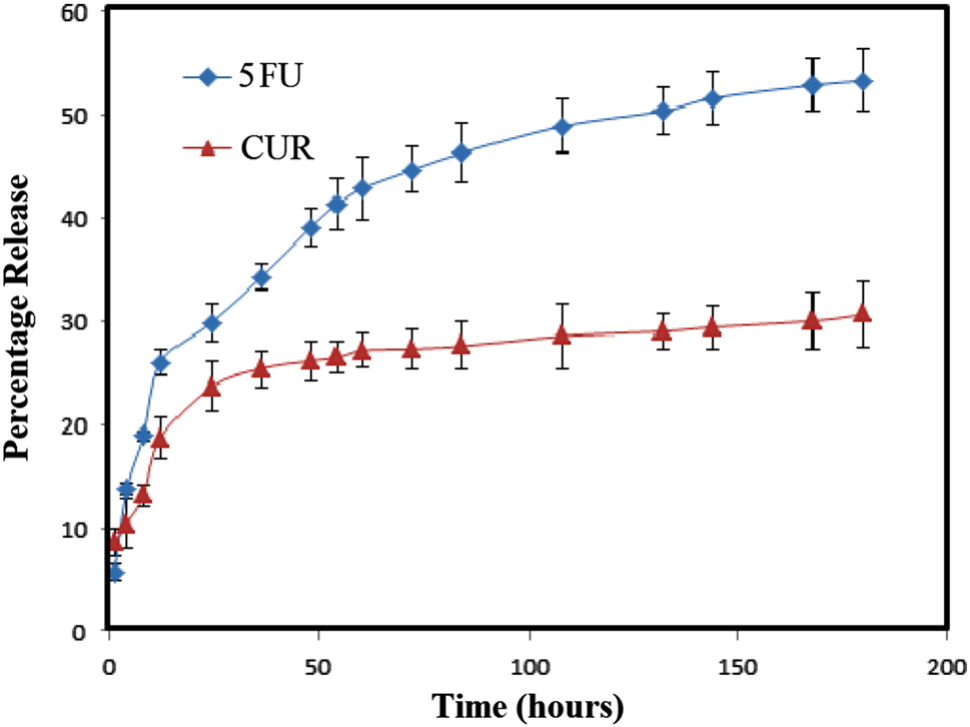
Drug release profiles of 5FU and CUR from 5FU-CUR-C-MNP-HSA-FA. *5FU* 5-fluorouracil, *CUR* curcumin, *5FU-CUR-C-MNP-HSA-FA* 5-fluorouracil and curcumin entrapment folic acid-decorated albumin-coated citrate-modified magnetic nanoparticles

To achieve nanoparticle-assisted combination tumor chemotherapy, the nano-sized vehicles should possess a property of controlled and sustained release with the ability to bind to a variety of drugs with five different physicochemical and therapeutic properties for simultaneous drug delivery (Singh and Lillard 2009). Curcumin typically binds to hydrophobic cavities of HAS (Kar et al. 2017), whereas 5FU binds to albumin by both hydrophobic interaction and hydrogen bonding (Chinnathambi et al. 2014). For 5FU, drug-loading capacity and entrapment efficiency were 1.9% and 19.1%, respectively. Similarly, drug-loading capacity and entrapment efficiency of 2.7% and 27.2% were noted for CUR. The drug release study of C-MNP-HSA-FA is presented in Fig. 6. It can be observed that, during the first 24 h, the release of 5FU was predominant and reached around 27%. Total release of around 54% was achieved in 200 h. Curcumin showed slow release profile compared to 5FU, with a total of 32% release over 260 h. Both drugs displayed a sustained release behavior, demonstrating that BSA-SPIOs would be excellent vehicles for dual drug loadings.

### In vitro cytotoxicity of drug-loaded NPs

**Fig. 7:**
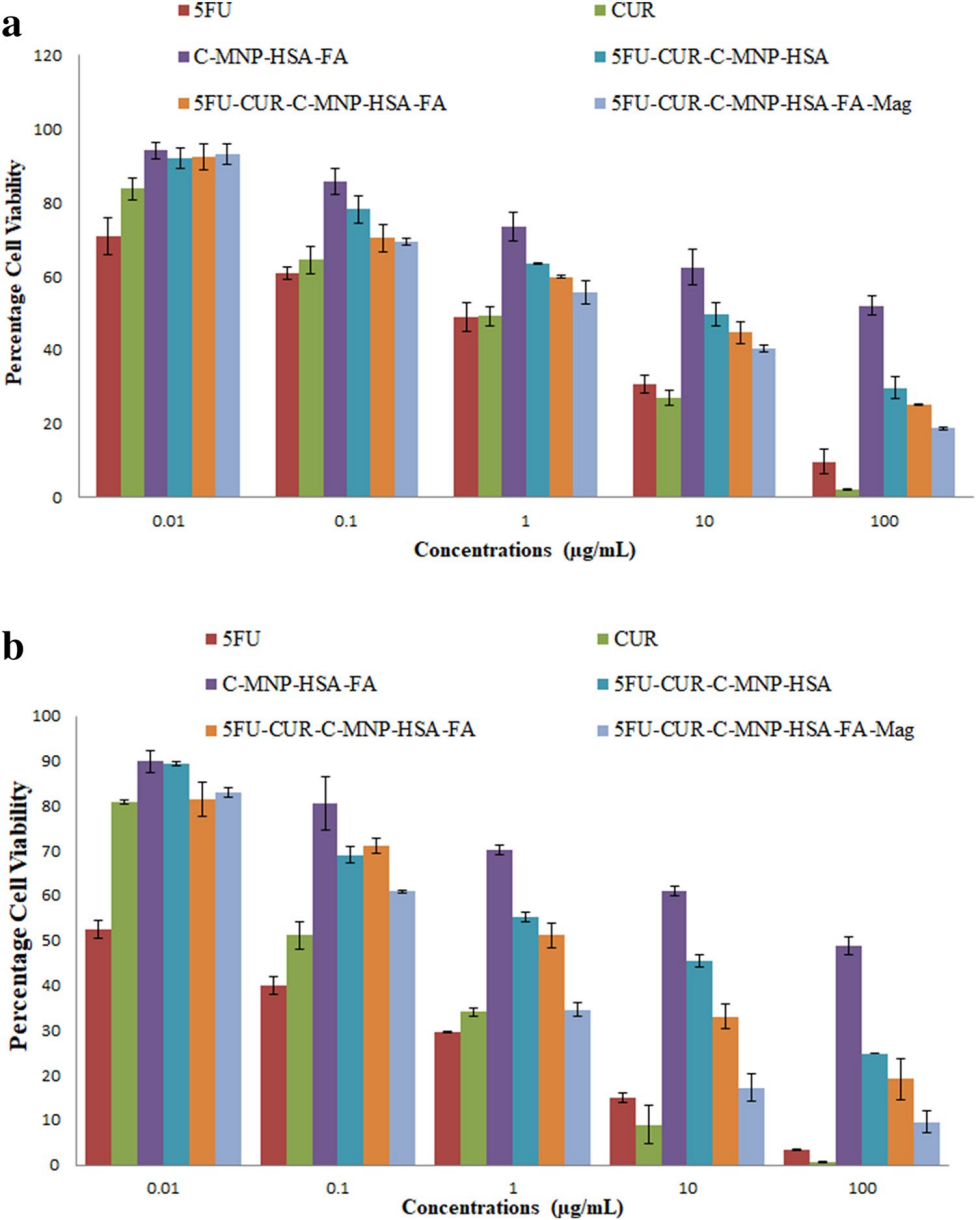
Cell viability of MCF-7 cells after incubation with 5FU, CUR, C-MNP-HSA-FA, 5FU-CUR-C-MNP-HSA, 5FU-CUR-C-MNP-HSA-FA, and 5FU-CUR-C-MNP-HSA-FA-mag for 48 h (**a**) and 96 h (**b**). *5FU* 5-fluorouracil, *CUR* curcumin, *C-MNP-HSA-FA* folic acid-decorated albumin-coated citrate-modified magnetic nanoparticles, *5FU-CUR-C-MNP-HSA* 5-fluorouracil and curcumin-loaded folic acid-decorated albumin-coated citrate-modified magnetic nanoparticles, *5FU-CUR-C-MNP-HSA-FA* 5-fluorouracil and curcumin entrapment folic acid-decorated albumin-coated citrate-modified magnetic nanoparticles, *5FU-CUR-C-MNP-HSA-FA-Mag* 5-fluorouracil and curcumin entrapment folic acid-decorated albumin-coated citrate-modified magnetic nanoparticles under magnetic field

MTT assay was performed under various time periods, 48 and 96 h, to assess cell viability of MCF-7 cells and results are presented in Fig. 7a, b. It is observed that the cytotoxicity of the drug-loaded nanoparticles is dependent on the drug concentration and the treatment period. Drug-loaded nanoparticles showed contrastingly lower values compared to C-MNP-HSA-FA which is obviously due to drug content. Furthermore, folic acid-conjugated nanoparticles showed higher cytotoxicity. IC_50_ values as determined by MTT assay of the 5FU, CUR, C-MNP-HSA-FA, 5FU-CUR-C-MNP-HSA, 5FU-CUR-C-MNP-HSA-FA, and 5FU-CUR-C-MNP-HSA-FA under magnetic field for 48 h were found to be 0.576, 0.409, 151.36, 6.567, 3.344, and 1.984, respectively. For 96 h, they were 0.184, 0.016, 96.34, 2.805, 1.191, and 0.340, respectively. Figure 7a, b shows the graphical depictions of cytotoxicity elicit by nanoparticles. Higher toxicity in case of folic acid-conjugated nanoparticles can be accredited to higher uptake by cells. Folic acid conjugation shows enhanced uptake by the cells, since folic acid binds to overexpressed folic acid receptors which lead to receptor-mediated endocytosis (Lin et al. 2013). Due to the higher cellular uptake of nanoparticles in contrast to non-targeted drug delivery systems, 5FU-CUR-C-MNP-HSA-FA shows higher uptake. In the presence of magnetic field, the cytotoxic effect of 5FU-CUR-C-MNP-HSA-FA was enhanced. In the presence of magnetic field, the concentration of nanoparticles near the surface of cells is higher, thus leading to higher uptake by the cells (Liu et al. 2012).

### Cellular uptake

**Fig. 8:**
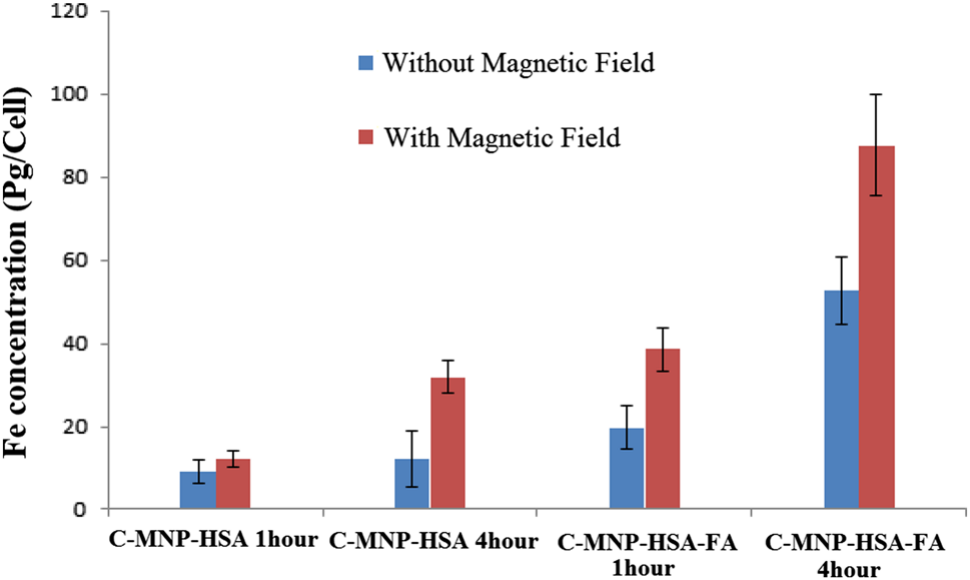
C-MNP-HSA, C-MNP-HSA-mag, C-MNP-HSA-FA, and C-MNP-HSA-FA-mag at 1 h and 4 h incubation. *C-MNP-HSA* albumin-coated citrate-modified magnetic nanoparticles, *C-MNP-HSA-FA* folic acid-decorated albumin-coated citrate-modified magnetic nanoparticles, *C-MNP-HSA-Mag* albumin-coated citrate-modified magnetic nanoparticles under magnetic field, *C-MNP-HSA-FA-mag* folic acid-decorated albumin-coated citrate-modified magnetic nanoparticles under magnetic field

Uptake of the nanoparticle is one of the important parameters for drug delivery applications. The nanoparticle uptake was analyzed by measuring iron content in the cell by AAS is as shown in Fig. 8. The iron content of approximately 9.16, 12.16, 19.8, and 52.77 pg/cell was witnessed by C-MNP-HSA, C-MNP-HSA-mag, C-MNP-HSA-FA, and C-MNP-HSA-FA-mag for 1 h incubation. Similarly, for 4 h incubation time, 12.16, 31.9, 38.6, and 87.6 pg/cell concentration was shown by C-MNP-HSA, C-MNP-HSA-mag, C-MNP-HSA-FA, C-MNP-HSA-FA-mag, respectively. Increased cellular uptake was noticed in case of 5FU-CUR-C-MNP-HSA-FA. Cell uptake in case of nanoparticles incubated for 1 h, folic acid conjugation brought about 1.32-fold increases in uptake, and the presence of the magnetic field further increased the uptake of nanoparticles by 1.9-fold. Similarly, 4 h incubation with folic acid-conjugated sample brought about 1.9-fold increases in cell uptake. Furthermore, 1.6-fold increases in uptake were brought about by a magnetic field. This increase is due to the receptor-mediated uptake of FA-conjugated nanoparticles in case of FA-conjugated samples and effect of the magnetic field that increases the concentration of nanoparticles at the cell surface.

## Conclusions

The results conclude that C-MNP-HSA-FA shows good aqueous suspending ability, and can be prepared and loaded with 5FU and CUR. Nanoparticles showed superparamagnetic behavior thus suitable for drug delivery applications. The controlled release of both the drugs was elicited by 5FU-CUR-C-MNP-HSA-FA. Drug-loaded nanoparticles showed a dose-dependent cytotoxic effect in MCF-7 cells. Enhanced cytotoxicity and cell uptake were observed in case of folic acid-conjugated nanoparticles, due to folic acid targeting. The external magnetic field can further enhance cytotoxicity and cellular uptake of nanoparticles due to magnetic targeting effect. Moreover, nanoparticles showed good magnetic saturation value and, hence, can be used to enhance treatment efficacy by magnetic targeting.
